# Growth performance, hepatic gene expression, and plasma biochemistry of rainbow trout fed full-fat meal, defatted meal, oil and chitin from black soldier flies

**DOI:** 10.1016/j.cirep.2024.200149

**Published:** 2024-05-23

**Authors:** M. Borland, C. Riesenbach, U. Shandilya, M.A. Chiasson, N.A. Karrow, D. Huyben

**Affiliations:** a Department of Animal Biosciences, University of Guelph, Guelph, ON, Canada; b Department of Biochemistry, Memorial University of Newfoundland, St. John's, NFLD, Canada; c Ontario Aquaculture Research Centre, Office of Research, University of Guelph, Elora, ON, Canada

**Keywords:** Immunity, Insects, Nutrition, qPCR, Salmonids

## Abstract

Insects, such as black soldier flies (BSF), are sustainable alternatives to fishmeal and fish oil that have been shown maintain fish growth performance, especially at low dietary inclusions. However, BSF is commonly processed to remove chitin and oil that may have additional benefits to the immune response and disease resistance of fish. The aim of this study was to investigate the hepatic gene expression and plasma biochemistry of rainbow trout (*Oncorhynchus mykiss*) fed BSF meals and BSF components. A three-month feeding trial was performed where rainbow trout (90.0 ± 4.0g; mean ± SD) were fed either a control diet or diets that contained: 10 % full-fat BSF, 10 % defatted BSF, 4 % BSF oil, and 1 % BSF chitin. Liver was analyzed for gene expression via qPCR and plasma was analyzed for 21 biochemical markers. The 4 % oil and 1 % chitin diets had the highest effect on growth performance, i.e. final weight (p=0.043) and feed intake (p=0.017), compared to the control diet, while no effects of these diets were found on hepatic gene expression. The 10 % full-fat BSF diet affected gene expression, specifically upregulating the pro-inflammatory cytokine *IL-8* (p=0.033), which may be due to the higher content of saturated fatty acids (SFA) and subsequently immune cell activity in the fish or the combination of the SFA and antimicrobial peptides in BSF that stimulate the innate immune system. No other immune and oxidative stress genes, or 21 plasma biochemical markers were found to be significantly affected (p>0.05). This research provides support to include and not remove oil and chitin from BSF since these components can improve growth performance and immune response of rainbow trout.

## Highlights

• Rainbow trout were fed black soldier fly (BSF) diets for three months.
• The 4 % oil and 1 % chitin BSF diets had the highest effect on growth performance.
• The 10 % full-fat BSF diet upregulated pro-inflammatory cytokine IL-8 in the liver.
• No effects of BSF diets on oxidative stress genes and 21 plasma biochemical markers.

## 1 Introduction

Rainbow trout (*Oncorhynchus mykiss*) is a freshwater salmonid species that is economically important and farmed in many countries, predominantly Chile, but also Norway, France, Italy, Spain, Denmark, USA, Canada, Germany, Iran, and the United Kingdom [1]. However, reduced use of antimicrobials and increased water temperatures have resulted in disease and high mortalities on fish farms [2]. Therefore, research on strategies to improve the production and health of rainbow trout is needed to maintain the growth of the aquaculture industry. In addition, alternatives to fishmeal and fish oil are needed to reduce pressures on fisheries and improve the environmental sustainability of the aquaculture industry [3]. In the past few decades, the depletion of wild fish stocks has led to higher inclusions of plant-based ingredients in aquafeeds [4]. However, plant ingredients such as soybean meal contain many anti-nutritional factors that can result in gut inflammation and enteritis, subsequently reducing growth performance of fish [5].

Insect-based ingredients, especially black soldier flies (*Hermetia illucens*; BSF), have been heavily researched in the past decade as alternatives to fishmeal and fish oil in aquafeeds. Several studies have found that up to 15 % inclusion in the diet does not reduce growth performance in Atlantic salmon (*Salmo salar*) and rainbow trout [6]. Aside from sources of protein and lipid, BSF has been found to have many immunomodulatory effects on fish as the protein fraction of BSF contains antimicrobial peptides, lipid fraction contains lauric acid that has antimicrobial properties, and chitin has immune-stimulatory effects [7,8]. BSF fed to rainbow trout was found to down-regulate intestinal *prostaglandin* and *interferon regulatory factor 1* genes, induce serum lysozyme activity, and prevent soybean meal-induced enteritis [9]. Another study found that feeding BSF to Atlantic salmon down-regulated expression of head kidney leukocyte antioxidants and stress-related genes with or without bacterial (LPS) and viral (Poly I:C) mimics [10]. Additional research found *heat shock proteins* (*HSP*) typically involved in regulating metabolic activity and immune signalling to be significantly down-regulated as a result of feeding lake whitefish (*Coregonus clupeaformis*) a 5 % full-fat BSF diet [11]. Another study observed activation of stress and immune response genes *tumor necrosis factor alpha* (*TNF-*α) and *IL-10* in the intestine of rainbow trout fed a 25 % and 50 % BSF inclusion diet [12]. However, there is a lack of knowledge on which components of BSF elicit these immunomodulatory properties and if they need both lipid and chitin in the BSF meal in order to have these effects. With these findings, this study aims to further understand the inflammatory and physiological responses in rainbow trout fed BSF.

We hypothesized that if the lipid fraction of BSF influences immune function, then feeding a 4 % BSF oil and 10 % full-fatted BSF diet will have a greater impact on hepatic gene expression of rainbow trout compared to the control, 1 % chitin and 10 % defatted BSF diets. The main objectives of this study were to perform a 3-month rainbow trout feeding trial with different components of BSF and analyze growth performance, hepatic gene expression and plasma biochemistry.

## 2 Materials and methods

### 2.1 Fish, facilities and diets

All procedures involving the handling and treatment of fish used in this study were approved by the Animal Care Committee at the University of Guelph under Animal Utilization Protocol #4743. Mixed sex rainbow trout (*Oncorhynchus mykiss*) averaging 90.02 ± 0.90 g in body weight (N=1260) were randomly distributed across 15 tanks (300 L) tanks at the Ontario Aquaculture Research Centre - Alma (Elora, ON, Canada) for three months (84 days). Fish were acclimated for three months in 120 L tanks prior to the feeding trial. A water flow-through system supplied each tank with 20 L/min of degassed groundwater and water quality was measured biweekly with an average temperature of 8.62 +/ 0.05 °C (mean ± SD) and dissolved oxygen of > 80 % saturation (8.7 mg/L). The experimental photoperiod was 12:12 light to darkness utilizing LED lights with a 60 min ramp time to stimulate dawn to dusk.

The tanks were set up in a randomized block design and triplicate tanks were fed one of five diets for 84 days. The diets were formulated and ground to be < 1 mm crumble to ensure proper mixing. Feed was prepared in the Department of Animal Biosciences, University of Guelph (Guelph, ON, Canada) by mixing for two 20 miin rounds, steam-pelleting (California Pellet Mill, USA) to 3 mm, drying overnight at 40°C and sieving to remove fine particles. The feed was stored at 4°C until the trial began.

During the feeding trial, the fish were hand fed to satiety twice per week. Feed was presented over a 2 hr period (9:00-11:00) in the morning and a 2 hr period (14:00-16:00) in the afternoon. For the remaining days, fish were fed using automatic belt feeders using a ration of 95 % of the average of the 2 previous hand-feeding days.

### 2.2 Sample collection

At start and end of the trial, all the fish were individually weighed and measured for fork length. Every 28 days, each tank was bulk weighed. At the end of the trial, 10 fish per tank were euthanized with an overdose of tricaine methanesulfonate (Syncaine®, Syndel Canada) and severing of the spinal cord. Liver and viscera were weighed to calculate body indexes. From 3 fish per tank, 1 cm of liver apex was collected into a sterile cryovial tube, snap frozen in liquid nitrogen, then store at -80°C. 3mL of blood was collected from the caudal vein using heparized syringes, centrifuged at 2,000 *g* for 2min and then plasma was decanted and frozen on dry ice.

### 2.3 Data calculations

The initial body weight of each fish was recorded at the start and end of the trial to calculate weight gain (WG), where WG= (Final body weight – Initial body weight) / Initial body weight * 100. Feed intakes (FI) were used to calculate the feed conversion ratio (FCR), where FCR = Feed intake (g) / Fish weight gain (g) on a wet matter basis. Weights of the viscera and liver were used to calculate the viscerosomatic index (VSI), where VSI = Viscera weight (g) / Body weight (g) *100 and hepatosomatic index (HSI), where HSI = Liver weight (g) / Body weight (g) *100).

### 2.4 RNA extraction and cDNA synthesis

A 30 mg piece of liver tissue from each fish was placed into a bead-beating tube containing 0.1 g of 1.0 mm silica beads (Sigma-Aldrich, Darmstadt, Germany) and 600 µL of RLT buffer (Qiagen, Toronto Canada). Next, the samples were homogenized in a Qiagen TissueLyzer II (Qiagen NV) for 2 miin at 30 Hz, left on ice for 2 miin, followed by homogenization for another 2 miin at 30 Hz. Once homogenization of the tissue was completed, 500 µL of the supernatant was removed and mixed with 500 µL of 70 % ethanol for bead removal. Next, 700 µL of the mixed ethanol and supernatant was placed into a spin column and centrifuged for 15 sec at 10,200 rpm at 4 °C using the microcentrifuge (Thermo Fisher Scientific, Waltham, MA, USA). Following this, 700 µL of RWI solution was added to the spin column and again centrifuged under the same conditions as mentioned previously. Samples were then treated with a DNase solution to remove any remaining contaminating DNA. This solution was formulated using 10 µL of the DNase with 70 µL of RDD buffer, which was centrifuged together and all 80 µL were pipetted into the spin columns and left to sit at room temperature for 15 miin. Following this, the mRNA was extracted using the RNeasy Mini Kit (Qiagen NV) according to the RNeasy Mini Kit Quick-Start protocol. Once all samples were extracted, RNA was quantified and assessed for quality using the BioTek Cytation-5, Cell Imaging Multi-Mode Reader (Agilent, Santa Clara, United States). Once ensuring all samples met the 260/280 nm absorbance ratio (OD ratio), cDNA synthesis was then performed.

The RNA samples were then diluted using nuclease-free water to obtain 1000 ng of RNA. The cDNA synthesis was performed using a high-capacity cDNA reverse transcription kit (Applied Biosystems, Waltham, United States) in 20 µL reaction volumes, comprising a 10 µL RNA sample, a 2 µL RT buffer, a 0.8 µL dNTP, a 2 µL random primer, 1 µL of reverse transcriptase and 4.2 µL of nuclease-free water according to the manufacturer's instructions. PCR conditions for cDNA synthesis included one cycle of 25 °C for 10 miin, 37 °C for 120 miin, and 85°C for 5 miin using the MiniAmp Thermo Cycler (Applied Biosystems). The cDNA samples were then diluted (1:5) to obtain 100 µL volumes.

### 2.5 Gene expression by RT-qPCR

All the candidate genes interrogated in this study were based on previous research that has found them to play an important role in diet and the immune response of rainbow trout. For each gene, the forward and reverse primers were selected, and listed in Table 2. The primer sequences were checked using BLAST (NCBI) and Primer3 (v.0.4.0), and gene products were verified using gel electrophoresis. Primers were diluted with nuclease-free water to a concentration of 10 µL. Next, duplicates of each sample were prepared and pipetted into individual wells of a 96-well plate in a 10 µL reaction comprising 1 µL cDNA, 6.4 µL nuclease-free water, 0.5 µL forward primer, 0.5 µL reverse primer and 5 µL SsoAdvanced Universal SYBR Green Supermix (Bio-Rad Laboratories Inc, Hercules, United States). Next, the PCR plates were centrifuged for 4 miin at 1500 rpm. RT-qPCR conditions maintained the samples first holding at 95 °C for 10 miin, then 40 cycles at 95°C for 15 sec, then 60 °C for 1 miin, followed by a melt curve at 95 °C for an additional 15 sec, 60°C for 1 miin, then 95°C for 15 sec using the StepOnePlus Real-time PCR system (Applied biosystems). This PCR protocol was altered slightly when performing qPCR on *hepcidin*, which displayed no specific bands during gel electrophoresis. As such, cDNA volume was increased from 2 to 3 μl and annealing temperatures were increased to 62 °C.

The reference genes chosen in this study included *β-actin* and *elongation factor 1 alpha* (*EF-1α*) as previous studies have indicated these genes to be stable sequences regardless of dietary treatment. However, when checking *β-actin* primer product through gel electrophoresis, no banding was identified. Therefore, this study used *EF-1α* as the only reference gene. The targeted genes were normalized using the geometric mean of the C_T_ values from the control diet: Δ C_T_ = Calibrator C_T_ – Sample C_T_. The fold-change (FC) was calculated as FC = (Primer Efficiency 2) ^ΔΔC_T_ relative to the reference gene.

### 2.6 Plasma biochemistry analyses

Plasma biochemistry analyses were performed by the Animal Health Laboratory at the University of Guelph using Cobas c501 module (Roche Diagnostic, Basel, Switzerland). 200 µl of plasma were pooled from 3 fish per tank and there were 3 tanks per diet (n=3 per diet). Plasma was analyzed by a mid volume analyzer comprised of a photometric unit for a broad range of clinical chemistry assays of total protein, albumin, globulin, glucose, cholesterol, aspartate aminotransferase, creatine kinase, amylase, lactate dehydrogenase, bile acid, glutamate dehydrogenase, carbon dioxide, lipase, uric acid and urea, and an ISE unit for ion-selective electrode determinations of sodium, potassium, chloride, calcium, and phosphorus.

### 2.7 Statistical analyses

When analyzing the gene expression data, all single Ct outliers were removed when the sample values equated to larger than [(standard deviation) *2 ± average] in each dietary group. The normal distribution and variance of homoscadicity of each dataset were determined using Shapiro-Wilk and Levene tests in RStudio version 1.3.1093. To normalize the data, log transformations were performed if needed. In this research, the p-values of each test diet were determined using the Least Square Means test with Tukey adjustment for multiple comparisons. Normal data were analyzed using the R function “aov” performing an ANOVA, which evaluated the effects of test diet on hepatic gene expression. Failing the assumptions required for ANOVA, a generalized linear model was performed on non-normal data. Here, a Kruskal-Wallis test was performed in addition to pairwise comparison using the Tukey Test for normal data, which used the p-value adjustment method of “BH” to correct for multiple testing. An “lme” was used as a linear effects model to include the random effect of fish tank, although it was not used since the Akaike Information Criterion (AIC) showed it did not improve the fit of the model to the data. A P-value < 0.05 was considered significant.

## 3 Results

### 3.1 Growth performance and relative body indices

Rainbow trout fed the 1 % chitin diet resulted in the highest growth in terms of final bodyweight (p=0.04) and feed intake (p=0.02), compared to the 10 % defatted BSF diet (Table 3). In addition, the 4 % BSF oil had a significantly higher final weight compared to the 10 % defatted BSF diet. The 10 % full-fat BSF and 4 % BSF oil diets did not negatively reduce growth parameters compared to the control diet. Both the VSI and HSI remained relatively normal with little variation among the test diets (p= 0.43 and p=0.28, respectively). The 10 % defatted BSF diet had the best (lowest) FCR, although not significant (p=0.06) and coincided with the lowest final weight.

### 3.2 Gene expression analysis

**Fig. 1:**
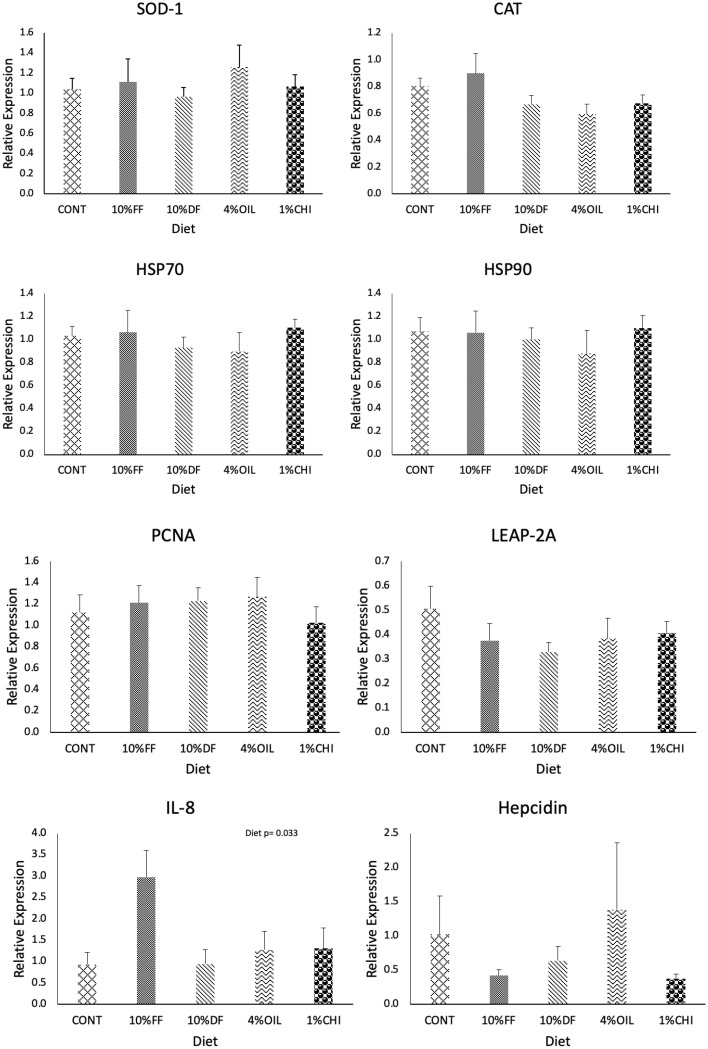
Hepatic gene expression relative to *EF-1α* (*elongation factor 1-alpha*) (mean ± SE) of rainbow trout fed a commercial control diet (CONT), a 10 % full-fat insect diet (10 %FF), a 10 % defatted insect diet (10 %DF), a 4 % oil inclusion insect diet (4 %OIL), and a 1 % chitin inclusion insect diet (1 %CHI) (*SOD-1: superoxide dismutase, CAT: catalase, HSP70: heat shock protein 70, HSP90: heat shock protein 90; PCNA: proliferating cell nuclear antigen, LEAP-2A: liver expressed antimicrobial peptide 2-A, IL-8: interleukin-8, and Hepcidin).* Calibrated using the control diet, where n=8 per diet.

Diet was not found to have any significant effects on the hepatic gene expression of *SOD-1, HSP70, HSP90, CAT, PCNA, LEAP-2A* and *hepcidin* (p > 0.05) (Fig. 1). However, diet had a significant effect on *IL-8* (p=0.03), where the pairwise comparison did not reveal statistical differences between the 10 % full-fat and 10 % defatted BSF diets (p=0.05 and p=0.05, respectively).

### 3.3 Plasma Biochemistry

Rainbow trout fed the 10 % full-fatted diet were found to have slightly elevated phosphorus and potassium levels compared to the rest of the diets (Table 5), however, this was statistically insignificant (p=0.54 and p=0.58, respectively). Additionally, rainbow trout fed the 4 % oil diet had elevated glutamate dehydrogenase levels compared to the other diets, however, this was also statistically insignificant (p=0.32). Feeding 1 % chitin increased plasma amylase and sodium concentrations, although not significantly (p=0.10 and p=0.08, respectively).

## 4 Discussion

The main objective of this study was to investigate the effects of feeding BSF meals and BSF components on growth performance, hepatic function, and plasma biochemistry in rainbow trout. This was investigated by measuring several parameters including weigh gain, feed conversion ratio, body indices, plasma biochemistry, and the hepatic gene expression of rainbow trout following a three-month feeding trial. This study generated important findings as insect-based diets have become more common as a sustainable feed option within the aquaculture sector, however, little is known about the impact of BSF meals and BSF components on growth performance and immune function in rainbow trout. This research aimed to improve the overall growth and health of rainbow trout while expanding the sustainability of the aquaculture industry.

### 4.1 Growth performance was highest when fed chitin

Rainbow trout fed the 1 % chitin diet resulted in the highest growth among all diets followed by the control, 4 % BSF oil, 10 % full-fat BSF and 10 % defatted BSF diets (Table 3). These results provide further evidence that feeding 10 % full-fat meal, 4 % oil and 1 % chitin from BSF can replace fishmeal and fish oil without negatively impacting the growth performance and FCR of rainbow trout. In agreement, a meta-analysis of 17 studies by Weththasinghe et al. (2021) fed BSF to rainbow trout and Atlantic salmon and found feeding between 5-60 % BSF in the diet or between 0-100 % replacement of fishmeal resulted in similar or improved growth performance compared to the control. Similar results have been found previously when BSF replaced animal-based protein in diets for Atlantic salmon that resulted in similar or improved growth and FCR [13]. In addition, another meta-analysis of 33 studies where several fish species were fed insects suggested a maximum inclusion of up to 29 % BSF [14]. In the present study, only 25 % of fishmeal was replaced in the 10 % full-fat BSF diet (Table 1), therefore, we did not expect or have any reductions in growth performance. However, fish fed the 10 % defatted BSF diet resulted in lower final weight, which may be due to the larger portion of fishmeal replaced, i.e. 38.5 % (Table 1). The combination of high soybean meal with the higher inclusion of insect may have resulted in lower growth performance, although more research is required. At least for the other diets, the results suggest providing rainbow trout with a low inclusion (10 %) of full-fat meal, oil and chitin from BSF can support the growth of rainbow trout, benefiting farm production.

**Table 1:** Diet formulations (g/kg) of a control (CONT) and BSF diets based on 10 % full-fat (10 %FF), 10 % defatted (10 %DF), 4 % oil (4 %OIL) and 1 % chitin (1 %CHI)

	CONT	10 %FF	10 %DF	4 %OIL	1 %CHI
*Ingredient (g/kg wet matter)*					
Fish Meal, herring	200	150	123	200	200
Poultry Meal	100	100	100	100	100
Wheat Meal	100	100	100	100	100
Fermented Soybean Meal	300	300	300	300	300
Full Fat BSF		100			
De-fatted BSF			100		
BSF Oil				40	
BSF Chitin					10
Wheat Gluten	50	50	50	50	50
Blood Meal	50	50	50	50	50
Corn Starch	50	35	32	50	40
Fish Oil, herring	67	32	62	27	67
Canola Oil	67	67	67	67	67
Vitamin Premix	5	5	5	5	5
Limestone	1	1	1	1	1
Salt	1	1	1	1	1
Choline Chloride	3	3	3	3	3
DL-Methionine	1	1	1	1	1
Vitamin E	5	5	5	5	5
*Proximate Composition (% dry matter)*					
Dry matter	94.0	94.3	94.1	93.7	94.0
Crude protein	45.4	46.1	44.3	43.3	45.7
Crude lipid	16.8	17.8	17.1	17.0	17.4
Ash	7.1	6.8	6.6	7.3	8.0
Carbohydrate*	24.6	23.7	26.0	26.1	22.9
Gross energy (MJ/kg)	21.8	22.4	22.1	21.7	21.6

⁎ Carbohydrate content determined based on dry matter minus protein, ash, and lipids BSF; black soldier fly.

**Table 2:** Forward and reverse primer sequences of target (pro- and anti-inflammatory cytokines, heat shock proteins, and oxidative stress) and reference genes, template size, annealing temperature, accession numbers, and sources references.

Target Gene	Forward Primer (5′-3′) Reverse Primer (5′-3′)	Size bp	Temp C	Accession	Reference
*Hepcidin*	GCTGTTCCTTTCTCCGAGGTGC GTGACAGCAGTTGCAGCACCA	165	60	AF542965	[15]
*Liver expressed antimicrobial peptide-2A (LEAP-2A)*	GGTTCCTGGTGTTTCTGGTGCT AGTGGCCACCCCTGCAAAT	222	60	AY362186	[15]
*Interleukin-8 (IL-8)*	CACAGACAGAGAAGGAAGGAAAG TGCTCATCTTGGGGTTACAGA	162	57	AJ279069	[16]
*Interleukin-10 (IL-10)*	TCACGTCATGAACGAGATCC CCTCTTGCATTTCACCATATCC	119	61	AB118099	[16]
*Heat shock protein 70 (HSP70)*	CCACTTCATCGCAGAGTTCAAA GCGAACAGCCCTCTTGTTGT	67	60	AB196460	[16]
*Heat shock protein 90 (HSP90)*	AGGGTCAAGGAGGTGGTCAA AACGAAGAGGGTGATGGGATATC	63	60	AB196457	[16]
*β-actin (β-act)*	GCGACCTCACAGACTACC ACAGTCCCATTGCTCCAGTCC	273	52	AB196465	[17]
*Elongation factor 1- α (EF-1α)*	CAACGATATCCGTCGTGGCA ACAGCGAAACGACCAAGAGG	159	60	AF498320	[15]
*Interleukin 1-B (IL-1B)*	TGCTGTGGAAGAACATATAGTG ACGAAGACAGGTTCAAATGC	212	60	AJ004821	[17]
*Proliferating cell nuclear antigen (PCNA)*	TGTGACCGCAACCTCGCAATGG CACGGCAGATACGGGCAAACTCC	265	60	CA359986	[17]
*Superoxide dismutase (SOD-1)*	CCACGTCCATGCCTTTGG TCAGCTGCTGCAGTCACGTT	141	60	BG936553	[18]
*Catalase (CAT)*	CCCAAGTCTTCATCCAGAAACG CGTGGGCTCAGTGTTGTTGA	101	60	BG935638	[18]

Fish fed with insect diets with higher lipid content (10 % full-fat, 5 % full-fat, and 4 % oil) showed greater final weight and feed intake when compared to lower lipid diets (10 % defatted). Dietary lipids are essential sources of energy, fatty acids, cholesterol and other functional metabolites for salmonids [19]. A study on Atlantic salmon found that higher levels of n-3 polyunsaturated fatty acids (PUFA), such as alpha linolenic acid (ALA), eicosapentaenoic acid (EPA) and docosahexaenoic acid (DHA), increased feed intake and fish growth [20]. BSF has high amounts of saturated fatty acids (SFAs), especially lauric acid, with subsequently low inclusions of n-3 PUFA, specifically small amounts of ALA and no EPA and DHA. Therefore, replacement of fish oil (high in n-3 PUFA) with BSF oils (low in n-3 PUFA) was expected to reduce growth performance of rainbow trout, which was not the case in this study since fish were able to grow optimally when fed BSF diets where the fish oil inclusion decreased from 6.7 to 2.7 % of the diet (Table 1). The n-3 PUFA derived from the inclusion of fish oil at 2.7 % of the diet and small amounts of n-3 PUFA from fishmeal, canola oil and other ingredients would supply the miinimum level of n-3 PUFA at 1.8 % of the diet (wet-matter basis) to meet the requirements for rainbow trout that weigh between 50-200 g [21]. Similar to our results, a study that fed defatted BSF at 7 and 13 % as well as BSF oil at 3, 5 and 10 % to rainbow trout found no significant effects on final body weight, weight gain, feed intake and HSI compared to the control diet [22]. Overall, our results agree with previous research that demonstrated salmonids have the ability to consume diets with higher SFA content derived from BSF without compromising growth performance.

Chitin is a polymer of N-acetyl-D-glucosamine and is primarily found in the exoskeletons of insects [23] and high levels of chitin in the diet have been found to impair the nutrient digestibility by binding to digestive enzymes [24,25]. The level of chitin in the BSF diets in the present study were probably not high enough to result in significant reductions in protein digestibility and consequently reduced growth performance. This agrees with the meta-analysis by Weththasinghe et al. (2021) that found BSF inclusions above 15-18 % in the diet resulted in reduced growth performance. An *in vitro* study that chemically digested six BSF and six mealworm meals found that crude protein digestibility was negatively correlated to chitin content in the insect meals [26]. The 1 % level of chitin used in the present study was based on a 10 % content of chitin in BSF found in previous studies as ADF [27,26], thus 1 % if we compared it to 10 % inclusion of defatted BSF meal in the diet. Despite the low protein digestibility of chitin, the low inclusion of 1 % chitin was not high enough to reduce growth performance and this low inclusion even stimulated growth, although not significantly (Table 3). These findings indicate BSF inclusion of 10 % or chitin inclusion of 1 % is not high enough to negatively impact growth performance.

**Table 3:** Means and P-values on growth performance of rainbow trout fed control (CONT) and BSF diets based on 10 % full-fat (10 %FF), 10 % defatted (10 %DF), 4 % oil (4 %OIL) and 1 % chitin for 3 months.

Parameter	CONT	10 %FF	10 %DF	4 %OIL	1 %CHI	SE	*p*-value
Final Weight (g/fish)	276	264	256	282	280	3.39	**0.043**
Weight Gain (g/fish)	186	176	171	186	187	2.52	0.127
Feed intake (g/fish)	167^b^	155^ab^	146^a^	164^ab^	170^b^	2.87	**0.017**
FCR	0.90	0.88	0.85	0.88	0.91	0.001	0.060
VSI	13.4	12.9	12.6	13.4	13.6	0.18	0.430
HSI	2.13	2.16	2.51	2.00	2.30	0.03	0.276

SE: pooled standard error; FCR: feed conversion ratio; VSI: viscerosomatic index; HSI: hepatosomatic index.

*Different lowercase letters show significant differences between dietary groups (P < 0.05). Bold number indicate significant effect (P < 0.05), where n=3 per diet using an ANOVA or Kruskal-Wallis test.

### 4.2 Gene expression only influenced by 10 % full-fat BSF

Only one out of eight target genes were significantly affected by diet in the present study (Table 4 and Fig. 1), which may be due to the lack of a pathogen challenge or stressor at the end of the study and long duration that allowed their immune system to adapt over time. In addition, change in the expression of a small handful of genes may not lead to changes in protein expression and immune response. Frenette et al. [28] found that changes in the expression of hundreds of genes only led to a 17-fold change in protein expression, thus more exploratory research possibly with RNA sequencing of the transcriptome is needed to determine the extent of these dietary effects. In addition, analysis of other tissues rather than the liver that are more active in the immune system, such as head kidney and spleen, are also recommended to improve the evaluation of the immune response in future studies. However, the numerical differences and one significant difference does reveal more information about the impact of BSF meals and BSF components on the immune response of rainbow trout.

**Table 4:** P-values for the effects of diet with varying levels of insect inclusion on relative gene expression in the liver of rainbow trout determined with an ANOVA or Kruskal-Wallis test (n=8).

Gene	Degrees of freedom	F-value	*p* -value
*SOD-1*	4	0.963	0.943
*CAT*	4	2.025	0.194
*IL-8*	4	0.960	**0.033**
*HSP70*	4	1.878	0.802
*HSP90*	4	0.855	0.866
*PCNA*	4	0.120	0.842
*LEAP-2A*	4	0.500	0.568
*Hepcidin*	4	0.718	0.879

Note: SOD-1: superoxide dismutase, CAT: catalase, IL-8: interleukin-8, HSP70: heat shock protein 70, HSP90: heat shock protein 90, PCNA: proliferating cell nuclear antigen, LEAP-2A: liver expressed antimicrobial peptide 2-A, and Hepcidin). P-values from linear fixed effects models with fixed effects of diet, protein, lipid, and insect. Bold number indicate significant effect (p < 0.05), where n=8 per diet.

Expression of *SOD-1* was found to be upregulated in diets containing 10 % full-fat and 4 % oil compared to the 10 % defatted, 1 % chitin and control diets, however not significantly (p=0.943) (Table 4 and Fig. 1). *Superoxide dismutase (SOD-1)* functions in response to detoxification and specifically acts as an indicator of oxidative stress [29]. In agreement, a previous study found that 85 % replacement of protein with BSF elevated the detoxification response in the Atlantic salmon by upregulating oxidative genes, e.g. *SOD-1* [18]. Similarly, a study was conducted to determine the effects of malic acid, an organic acid studied for its effects on gut health, which found significant increases in serum *SOD* activity compared to normal dietary conditions in rainbow trout [30]. The present study aimed to investigate insect-based feed additives to improve detoxification activity in order to reduce oxidative stress in rainbow trout. Several theories exist regarding the promotion of antioxidant activity in the gut of rainbow trout following consumption of insect-based diets. For example, one study suggested that higher antioxidant activity was correlated with the heavy metals present in BSF diets that are accumulated when insects are cultured in metal-enriched substrates [18]. Heavy metals accumulate predominately in the oil [31], which is supported by upregulation, of *SOD-1* in the liver of fish fed BSF ingredients that included BSF oil, however, not statistically significant (p=0.943) (Table 4 and Fig. 1). In agreement, a study that fed 12 % full-fat BSF larvae to largemouth bass (*Micropterus salmoides*) found that expression of *SOD* was upregulated compared to the control diet, but not significantly [32]. In addition, *catalase (CAT)* was also used in this study as a target gene to indicate increased detoxification (oxidative stress) in rainbow trout and the results showed an upregulation of *CAT* in rainbow trout fed the 10 % full-fat BSF and 4 % BSF oil diets, although not significant (p=0.194) (Table 4 and Fig. 1). A previous study that replaced 85 % of protein with BSF found that *CAT* was upregulated and suggested that this inclusion rate of BSF was too high and could impair fish health [18]. Overall, this study supported previous research and that 10 % full-fat BSF and 4 % BSF oil diets did not significantly effect the detoxication activity and oxidative stress of rainbow trout, while higher inclusions may be at risk of causing these ill effects.

Expression of heat shock proteins (*HSP70* and *HSP90*) were slightly downregulated when fish were fed the 10 % full-fat BSF and 4 % BSF oil diets, although not significantly (p=0.802 and 0.806) (Table 4 and Fig. 1). *HSPs* have been widely studied to protect cells from the potentially damaging effects involved with stress including heat shock, hypoxia, ischemic/reperfusion injury, and exposure to environmental contaminants [33]. Previous research has indicated downregulation of *HSP* indicates a positive effect on rainbow trout since fewer cells need repair [16]. In agreement, a previous study that fed an 85 % BSF diet to Atlantic salmon found no effects on HSP gene expression compared to the control diet [34]. The absence of effected gene expression related to cellular repair when fed BSF further supports the use of BSF in rainbow trout diets. More research is needed on the ability of full-fat BSF and BSF oil, potentially at higher inclusion levels, to downregulate the expression of *HSP70* and *HSP90* since this may reduce overall cellular stress and repair in rainbow trout.

*Hepcidin* and *liver-expressed antimicrobial peptide 2A (LEAP-2A)* are liver-specific antimicrobial peptides investigated during this trial to determine if they were affected by insect-based diets. *Hepcidin* is an antimicrobial peptide that responds to pathogen challenges, typically functioning against bacteria, fungi, and viruses [35,36]. In this study, *hepcidin* was highly upregulated in the 4 % oil, however, both *hepcidin* and *LEAP-2A* were non-significantly downregulated in all other test diets when compared to the control diet (Table 4 and Fig. 1). In previous studies that identified genes upregulated as a result of induction of cortisol (stress) treatments in rainbow trout, it was noted that mRNA abundance of *hepcidin* was suppressed in the liver [15]. In addition, genetic research found *hepcidin* was required during antimicrobial responses since its metal-binding site at the N-terminus is involved in oxidative damage against macromolecules [37]. Reduced expression of *hepcidin* may indicate that BSF protein and chitin could be an effective way to reduce stress and pathogenic challenges in rainbow trout. The 4 % BSF oil diet caused a non-significant upregulation of *hepcidin*, however, previous research has shown insect oil contains greater SFA content and specifically high levels of lauric acid that has antimicrobial properties [34], which is not in agreement with upregulation of the antimicrobial peptide *hepcidin. LEAP-2A* functions similarly to *hepcidin*, which is involved in activities against bacterial, fungal, and viral pathogens [15]. Researchers describe a positive result when *LEAP-2A* is downregulated as a result of increased cortisol, suggesting stress pathways suppress lipopolysaccharide abundance in the liver [15]. Overall, this requires further research to determine the impact high BSF oil inclusion has on *hepcidin* and *LEAP-2A* genes involved in antimicrobial activity.

*Interleukin-8 (IL-8)* is a chemokine or pro-inflammatory cytokine, which when upregulated, is indicative of activated inflammatory pathways and a heightened innate immune response [16]. In this study, *IL-8* was upregulated in fish fed the 10 % full-fat test diet (p=0.033) (Table 4 and Fig. 1). These results suggest the high insect lipid inclusion in the 10 % full-fat BSF diet caused negative effects on lipid metabolism which has been found in previous research [38]. The n-3 PUFA's, such as EPA, are precursors to eicosanoids and other molecules involved in the innate immune response [39], thus replacement of fish oil high in PUFA's with insect components low in n-3 PUFA would reduce the immune cells and activity. A study that fed low levels of n-3 PUFA to Atlantic salmon under normoxia found increased production of eicosanoids and upregulation of *IL-1β* [40], possibly to compensate for low n-3 PUFA in the diet. A study that fed 11 and 21 % full-fat BSF to rainbow trout resulted in a higher ratio of SFA to UFA compared to the control as well as a higher fat accumulation in the liver for fish fed the 21 % full-fat BSF diet [38]. *Proliferating cell nuclear antigen (PCNA)* was also used to indicate DNA repair and inflammation in salmonids [41,17]. In the present study, *PCNA* expression was not affected, indicating no severe DNA repair and inflammation in the liver of fish rainbow trout. These findings suggest that the 10 % full-fat BSF diet may be inducing unnecessary inflammation by lowering the n-3 PUFA levels in rainbow trout, however, more research is needed to understand why similar results were not found when fish were fed the 4 % BSF oil diet.

Previous literature indicated chitin acts as a beneficial prebiotic substrate for probiotic bacteria in the gut of rainbow trout leading to higher alpha diversity (e.g. Chao-1 Richness) that benefits the host [16]. Harikrishnan et al. [42] found that the inclusion of a 1 % chitin and chitosan diet significantly impacted the hematology and immune response in Kelp grouper (*Epinephelus bruneus*), specifically red blood cell counts, hemoglobin levels, and counts of lymphocytes, monocytes, and neutrophils [43]. These changes in leukocyte numbers have been largely attributed to the indirect effects of the gut microbiome that feed off chitin and chitosan, and interact with the host immune system [44]. In addition, a study that fed an untreated BSF diet to Atlantic salmon found that the gut bacteria had increased diversity and evenness of the community compared to the salmon fed dechitinated and fermented BSF diets [13]. As such, it was expected that the 1 % chitin diet as well as the other BSF diets, except 4 % BSF oil, would have extensively modulated gene expression. However, similar effects of antimicrobial fatty acids (e.g. lauric acid) in the BSF oil and antimicrobial peptides in the BSF protein may be interfering with beneficial effects on the immune and oxidative stress pathways. Most of the target genes selected in the present study were from previous studies that investigated single stimulants, thus beneficial effects of different BSF components may have been missed and more exploratory methods are needed to determine the effects on these pathways.

### 4.3 No effects of BSF on plasma biochemistry

Plasma biochemistry parameters overall remained relatively unaffected by BSF inclusion in the diet (Table 4), which indicates no negative effects of BSF and BSF components on rainbow trout, at least at the levels used in the current study. Aspartate aminotransferase (AST), an indicator of disease and liver dysfunction [45], was not found affected the diet (p=0.780), however, the 10 % full-fat diet had slightly higher (non-significant) concentrations of AST compared when compared to 10 % defatted diet. Furthermore, CK concentration was not significantly impacted by the BSF diets (p=0.970; Table 5). CK is found concentrated in muscle and heart tissues and can be an indicator of damage to the tissue (Weththasinghe et al., 2021), thus muscle and heart tissues of rainbow trout fed BSF and BSF components were healthy in the present study. Weththasinghe et al. (2021), supported this research when finding CK levels to remain unaffected in Atlantic salmon fed a 20 % full-fat and 15 % defatted BSFL diet. In this research, the 10 % full-fat diet had slightly higher levels of CK (non-significant) in the plasma biochemistry when compared to the control diet. BSF in the present study did not affect glutamate dehydrogenase levels in the plasma of rainbow trout and this was also found in studies that fed 5 % full-fat BSF to lake whitefish [11]. The BSF diets in the present study were formulated with similar protein levels which resulted in the total protein and urea in the plasma analysis remaining unaffected (p=0.836 and 0.646, respectively). In contrast, a previous study found feeding 30 % oil from BSF elevated blood urea concentrations in barramundi (*Lates calcarifer*), while the present study used a substantially lower inclusion level of 4 % oil from BSF [7]. Lastly, fish fed the 1 % chitin had slightly higher (non-significant; p=0.096) plasma amylase (p=0.096) and sodium (p=0.084) concentrations, although this can be explained by a lower carbohydrate and higher ash content in the 1 % chitin diet (Table 1). These findings suggest that feeding 10 % full-fat, 10 % defatted, 4 % oil and 1 % chitin from BSF had no negative effects on plasma biochemistry and the health of rainbow trout, although borderline levels of AST, CK and urea caution higher levels of BSF inclusion are not recommended in the diet for rainbow trout.

**Table 5:** Means and p-values for the effects of diet, protein, lipid levels, and insect inclusion on the plasma biochemistry parameters of rainbow trout.

**Parameter**	**CONT**	**10 %FF**	**10 %DF**	**4 %OIL**	**1 %CHI**	**SE**	***p*-value**
Calcium (mmol/L)	2.61	2.47	2.53	2.53	2.68	0.04	0.471
Phosphorus (mmol/L)	5.05	5.35	4.48	4.80	4.80	0.20	0.541
Total Protein (g/L)	32.3	31.7	30.0	30.7	31.7	0.63	0.836
Albumin (g/L)	16.7	16.7	15.3	16.0	16.3	0.31	0.775
Globulin (g/L)	15.7	15.0	14.7	14.7	15.3	0.40	0.941
AG Ratio	1.07	1.11	1.05	1.11	1.07	0.02	0.926
Glucose (mmol/L)	4.00	3.77	4.50	4.43	4.40	0.20	0.790
Cholesterol (mmol/L)	8.05	7.41	7.01	6.98	8.01	0.24	0.475
Aspartate aminotransferase (AST; U/L)	579	938	531	623	506	108	0.778
Creatine Kinase (CK; U/L)	10090	35961	6245	17758	9839	6159	0.970
Amylase (U/L)	643	675	556	529	715	26.4	0.096
Lipase (U/L)	5.67	5.00	5.00	5.33	5.33	0.15	0.623
Uric Acid (μmol/L)	38.7	26.7	28.0	25.7	24.7	2.35	0.348
Lactate Dehydrogenase (U/L)	2207	4061	1725	2387	1770	713	0.994
Bile Acid (μmol/L)	23.0	16.0	13.7	29.3	10.3	2.67	0.141
Glutamate Dehydrogenase (GLDH; U/L)	122	146	140	163	95.7	10.3	0.220
Sodium (mmol/L)	152	148	150	151	154	0.56	0.084
Potassium (mmol/L)	4.63	6.20	4.60	4.87	4.20	0.36	0.577
Chlorine (mmol/L)	121	117	120	121	122	0.82	0.461
Carbon Dioxide (mmol/L)	10.0	10.7	10.7	10.7	10.0	0.16	0.406
Urea (mmol/L)	0.70	0.67	0.63	0.73	0.60	0.03	0.646

10 %DF; 10 % defatted BSF, 10 %FF; 10 % full-fat BSF, 1 %CHI; 1 % chitin BSF, CONT; Control, 4 %OIL; 4 % oil BSF

*Bold number indicate significant P-value < 0.05. No significant results were found for a lipid*protein interaction, where n=3 for each diet.

## 5 Conclusions

In conclusion, our hypothesis was supported in that diets with insect oil, specifically, 10 % full-fat BSF and 4 % BSF oil had higher impacts on the chemokine *IL-8* and oxidative stress in the liver of rainbow trout compared to the control, 1 % chitin and 10 % defatted BSF diets. Our results also showed final weight and feed intake of rainbow trout was improved when fed diets with 4 % BSF oil, although only compared to the 10 % defatted BSF diet and not the control diet. No effects on FCR, VSI, HSI and 21 plasma biochemistry parameters were found between diets, which indicated BSF inclusion had no negative effects on the health of rainbow trout. We found that feeding 10 % full-fat BSF diet upregulated the gene expression of *IL-8*, which may be due to the higher content of SFA and subsequently immune cell activity in the fish or the combination of the SFA and antimicrobial peptides in BSF that stimulate the innate immune system. The 4 % BSF oil diet also upregulated expression of *SOD-1* and *hepcidin*, although not significantly, but may indicate effects on oxidative stress and gut microbiome responses. Overall, we recommend the 10 % full-fat BSF diet for rainbow trout based on the maintained growth parameters and potentially positive effects on hepatic gene expression. Additionally, we also recommend the 1 % chitin diet for rainbow trout due to the highest final weight and feed intake despite the lack of effect on gene expression. Further effects of BSF chitin and oil should be investigated since only eight commonly studied genes were investigated in this study, thus more exploratory techniques, such as RNA sequencing, need to be used to better understand alternative gene expression pathways. These findings from this study are significant since they demonstrate other components of BSF, such as BSF oil and chitin, can be effective ingredients in diets for rainbow trout without causing negative effects on growth performance and potentially beneficial effects on the immune response that can assist in improving fish production and disease resistance.

## Funding

This study was funded by the 10.13039/501100000094Ontario Ministry of Agriculture, Food and Rural Affairs (OMAFRA) Alliance Tier I (UG-T1-2021-101077) and 10.13039/501100000038Natural Sciences and Engineering Research Council of Canada (NSERC) Alliance (ALLRP-2021-568553-21). Special thanks to Enterra Feeds and Bluewater Feed (Sharpe Farm Supplies) for providing the ingredients.

## CRediT authorship contribution statement

**M. Borland:** Writing – original draft, Investigation, Formal analysis, Data curation. **C. Riesenbach:** Writing – review & editing, Investigation, Formal analysis. **U. Shandilya:** Writing – review & editing, Investigation, Formal analysis. **M.A. Chiasson:** Writing – review & editing, Resources, Conceptualization. **N.A. Karrow:** Writing – review & editing, Resources. **D. Huyben:** Writing – review & editing, Supervision, Project administration, Investigation, Funding acquisition, Conceptualization.

## Declaration of competing interest

The authors declare that they have no known competing financial interests or personal relationships that could have appeared to influence the work reported in this paper.

## Data Availability

Data will be made available on request.
